# Higher-Order Thalamic Encoding of Somatosensory Patterns and Bilateral Events

**DOI:** 10.3389/fncir.2021.752804

**Published:** 2021-10-25

**Authors:** Carlos Castejon, Jesus Martin-Cortecero, Angel Nuñez

**Affiliations:** ^1^Department of Anatomy, Histology and Neuroscience, Autónoma de Madrid University, Madrid, Spain; ^2^Institute of Physiology and Pathophysiology, Medical Biophysics, Heidelberg University, Heidelberg, Germany

**Keywords:** thalamocortical (TC), corticothalamic circuit, whisker, POm, bilateral, sensory patterns, thalamus

## Abstract

The function of the higher-order sensory thalamus remains unclear. Here, the posterior medial (POm) nucleus of the thalamus was examined by *in vivo* extracellular recordings in anesthetized rats across a variety of contralateral, ipsilateral, and bilateral whisker sensory patterns. We found that POm was highly sensitive to multiwhisker stimuli involving diverse spatiotemporal interactions. Accurate increases in POm activity were produced during the overlapping time between spatial signals reflecting changes in the spatiotemporal structure of sensory patterns. In addition, our results showed for first time that POm was also able to respond to tactile stimulation of ipsilateral whiskers. This finding challenges the notion that the somatosensory thalamus only computes unilateral stimuli. We found that POm also integrates signals from both whisker pads and described how this integration is generated. Our results showed that ipsilateral activity reached one POm indirectly from the other POm and demonstrated a transmission of sensory activity between both nuclei through a functional POm-POm loop formed by thalamocortical, interhemispheric, and corticothalamic projections. The implication of different cortical areas was investigated revealing that S1 plays a central role in this POm-POm loop. Accordingly, the subcortical and cortical inputs allow POm but not the ventral posteromedial thalamic nucleus (VPM) to have sensory information from both sides of the body. This finding is in agreement with the higher-order nature of POm and can be considered to functionally differentiate and classify these thalamic nuclei. A possible functional role of these higher-order thalamic patterns of integrated activity in brain function is discussed.

## Introduction

Traditionally, sensory systems have mostly been studied using simple and discrete stimuli. However, in natural conditions, sensory events usually have complex and dynamical spatiotemporal structures and normally multiple sensory signals occur simultaneously with different onsets, offsets, and overlappings, challenging the computational capacities of sensory systems. However, it is still unclear how these systems generate a representation of these dynamics. Moreover, how sensory systems extract relevant patterns from the raw sensory flow is poorly understood. Here, we propose the hypothesis that higher-order sensory thalamus could have a fundamental role in that function.

The rodent whisker system has an extraordinary ability to extract patterns and regularities from the environment and provides a perfect model in which to test our proposal. Rodents have an array of whiskers on each side of the face and during tactile exploration, multiple whiskers are stimulated simultaneously. Accordingly, the activation of individual whiskers strongly overlaps. These multiple contacts with the whiskers generate different patterns of sensory information. How the somatosensory system transforms these merged raw sensory signals into reliable neural representations and extracts information from that apparent noise is incompletely understood.

In these animals, tactile information from whiskers is processed by parallel ascending pathways toward the cortex (Chiaia et al., 1991; Diamond et al., 1992; Sherman and Guillery, 1998; Ahissar et al., 2000; Veinante et al., 2000a; Ohno et al., 2012; Clascá et al., 2016). The ventral posteromedial thalamic nucleus (VPM) and the posteromedial thalamic nucleus (POm) are implicated in these pathways. Although the function of VPM has been broadly studied, less is known about the function of POm. In contrast to VPM, POm neurons are characterized by large multiwhisker receptive fields (Chiaia et al., 1991; Diamond et al., 1992; Castejon et al., 2016). The functional implication of this characteristic of POm remains unclear.

In addition, also in contrast to VPM, POm projects to primary and higher-order cortical areas (Theyel et al., 2010; Ohno et al., 2012; Clascá et al., 2016; Casas-Torremocha et al., 2019; Zhang and Bruno, 2019; El-Boustani et al., 2020) and receives cortical driver input from layer 5 of these areas (Veinante et al., 2000b; Theyel et al., 2010; Groh et al., 2014; Mease et al., 2016). Accordingly, POm is well positioned to encode higher-order information of sensory, motor and associative nature. However, the content of POm representations and the nature of the messages that POm transfers to and receives from the cortex remain unclear.

Importantly, sensory events are usually characterized by bilateral sensory patterns. Therefore, the integration of tactile information from the two sides of the body seems to be fundamental in the coding of sensory patterns in bilateral perceptual function. Although, somatosensory cortical implication in the processing of bilateral stimuli has been much more studied (Armstrong-James and George, 1988; Shuler et al., 2001; Debowska et al., 2011), the role of the thalamus in these tactile bilateral interactions remains unknown.

The following experiments were thought to study the implication of POm in the encoding of these phenomena. Our results show for first time that POm is also able to respond to tactile stimulation of ipsilateral whiskers. We found that POm constantly integrates bilateral sensory information and that ipsilateral activity reaches POm *via* corticofugal projections mostly from S1. This in agreement with previous findings showing the convergence of ascending driver inputs from the periphery and descending driver inputs from L5 of S1 in POm (Groh et al., 2014; Castejon et al., 2016). In our study, we took advantage of the fact that ipsilateral sensory stimulation produced the activation of these corticothalamic fibers to investigate the functional interaction of these two streams in the codification of bilateral events. Our observations reveal a different implication of VPM and POm in bilateral perception. This finding is in agreement with the higher-order nature of POm and can be considered to functionally classify these thalamic nuclei.

## Materials and Methods

### Ethical Approval

All experimental procedures involving animals were carried out under protocols approved by the ethics committee of the Autónoma de Madrid University and the competent Spanish Government agency (PROEX175/16), in accordance with the European Community Council Directive 2010/63/UE.

### Animal Procedures and Electrophysiology

Experiments were performed on adult Sprague Dawley rats (220–300 g) of both sexes (40 males and 56 females). Animals were anesthetized (urethane, 1.3–1.5 g/kg i.p.) and placed in a Kopf stereotaxic frame. Local anesthetic (Lidocaine 1%) was applied to all skin incisions. The skull was exposed and openings were made to allow electrode penetrations to different neuronal stations in the trigeminal complex, thalamus and cortex.

**FIGURE 1:**
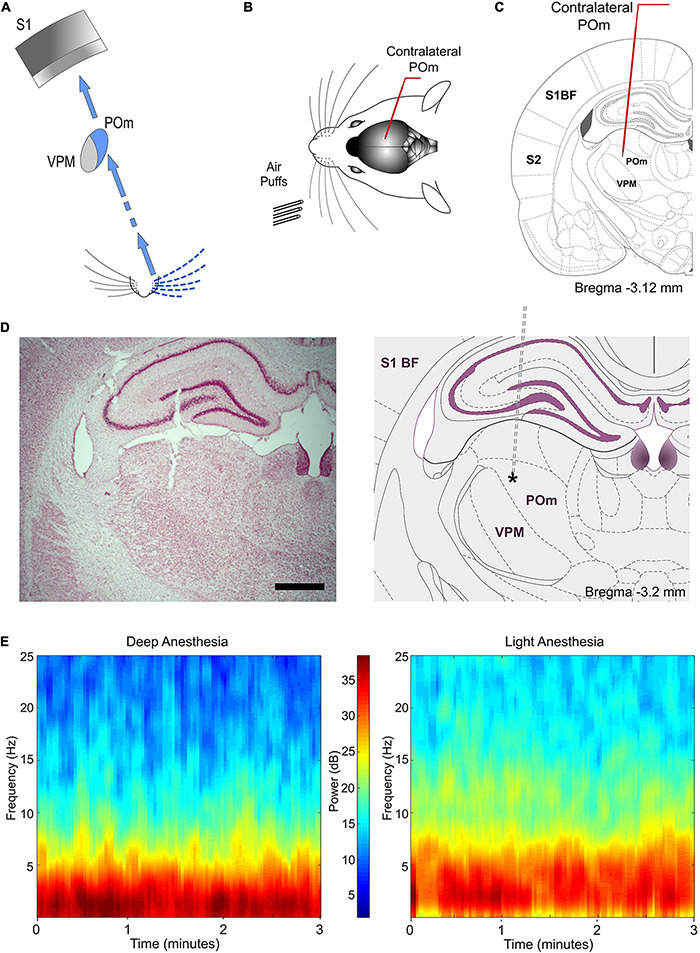
Experimental paradigm. **(A)** Illustration of the paralemniscal pathway. **(B)** Schematic drawing displaying the sensory stimulation *via* patterns of multiwhisker deflections. Recordings were made in the contralateral POm **(C)**. Coronal section illustrating a recording electrode inserted into POm. Bregma anteroposterior level is indicated. **(D)** The left panel shows a representative Nissl stained coronal section displaying the location of the recording site in the dorsolateral part of POm and the track left by the electrode. An atlas schematic reconstruction of this recording site within POm is shown in the right panel (Paxinos and Watson, 2007). Tip position is indicated by an asterisk. Scale bar, 1 mm. S1 BF, primary somatosensory cortex barrel field. S2, secondary somatosensory cortex. **(E)** POm LFP spectrograms of both deeply and lightly anesthetized states. Power spectra dominated by 1–2 Hz were only observed during deep anesthesia (Friedberg et al., 1999). Our recordings were mostly performed in the lightly anesthetized state.

Our recordings were performed several hours after the application of urethane (typically after 5–6 h). During the first hours after the application of urethane power spectra were dominated by 1–2 Hz (deeply anesthetized state). However, after 5–6 h, the level of anesthesia decreased and power spectra shifted indicating a lightly anesthetized state (Figure 1E; Friedberg et al., 1999). Our recordings were performed in this lightly anesthetized state. Supplementary doses of urethane were applied in those cases in which the level of anesthesia excessively decreased allowing the appearance of whisker movements. The body temperature was monitored and maintained at 37°C with a heating pad.

Extracellular recordings were performed using single microelectrodes in the Principal (PrV; Posterior from bregma 9–10; Lateral from midline 3–3.5, Depth 8.5–9.5; in mm) and Interpolar trigeminal nuclei (SpVi; P 11.5–14; L 2.5–3.5, D 8.5–9.5) of the trigeminal complex, in the posteromedial thalamic nucleus (POm; P 2.5–4.5, L 2–2.5, D 5–6.5), in the ventral posteromedial thalamic nucleus (VPM; P 2.8–4.6, L 2–3.5, D 5.5–7), and in the vibrissal region of the primary somatosensory cortex (S1; AP 0.5–4, L 5–7). Laminar recordings in supra- (D 150–550 μm), granular (D 650–850 μm), and infragranular (D > 950 μm) layers of S1 were also performed. Unanalyzed gaps were left between layers to compensate for differences in cortical thickness across this area and as a safeguard against potential errors in laminar localization. Tungsten microelectrodes (2–5 MΩ) were driven using an electronically controlled microdrive system (David Kopf).

### Sensory Stimulation and Patterns Generation

**FIGURE 2:**
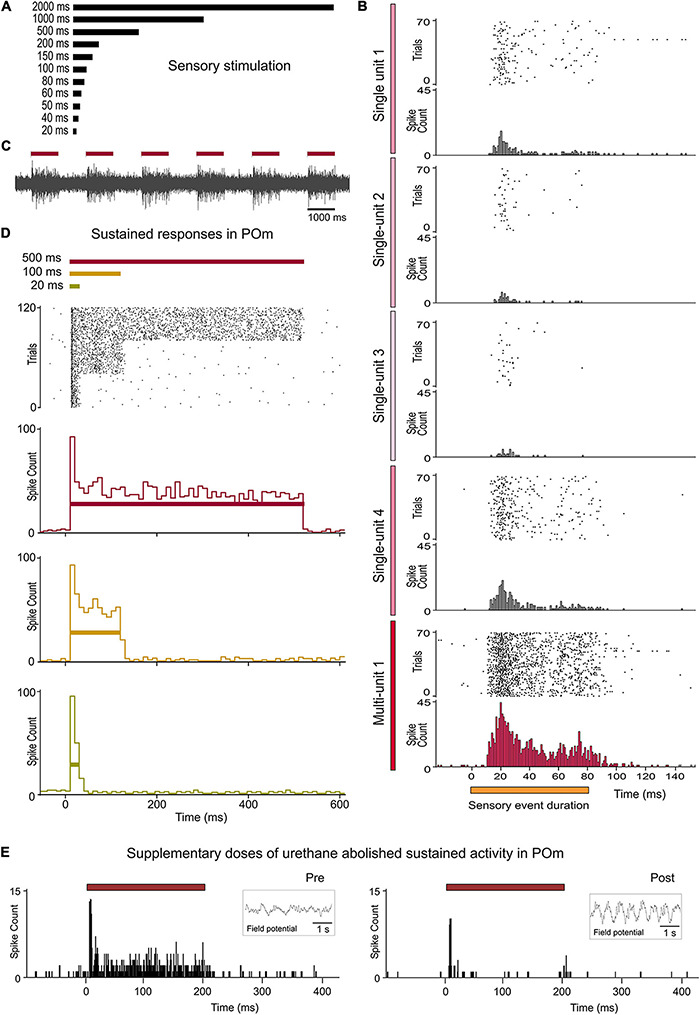
Sustained activity in POm. **(A)** Air-puffs used for sensory stimulation varied in duration from 20 ms to 2 s. **(B)** The encoding of the duration of sensory events by POm was studied at single-unit and multi-unit level. Raster plots and PSTHs showing one multi-unit and four single-unit POm responses extracted from the same recording and evoked by a sensory pattern of 80 ms duration. Note that the robustness of this form of encoding by POm sustained activity is obtained from population response formed by the superposition of spikes from individual neurons. The gradual color intensity of vertical lines represents a simulated contribution of each single-unit in this example to the encoding of stimulus duration. **(C)** Example recording in POm showing evoked sustained activity lasting the duration of the stimulus. **(D)** POm responds throughout the entire duration of the stimuli. Raster plot and PSTHs (bin width 10 ms) showing sustained multi-unit POm responses evoked by different stimulus duration (40 trials shown for each stimulus). Note that the duration of the stimulus did not alter the onset latency of responses. **(E)** POm sustained responses were highly affected by the level of anesthesia. They were only observed during the lightly anesthetized state. Supplementary doses of urethane abolished sustained activity in POm. Field potential activity recorded in each condition is shown in the insets. Horizontal color lines indicate the duration of the stimulus. Time 0 indicates the onset of the stimulus.

**FIGURE 3:**
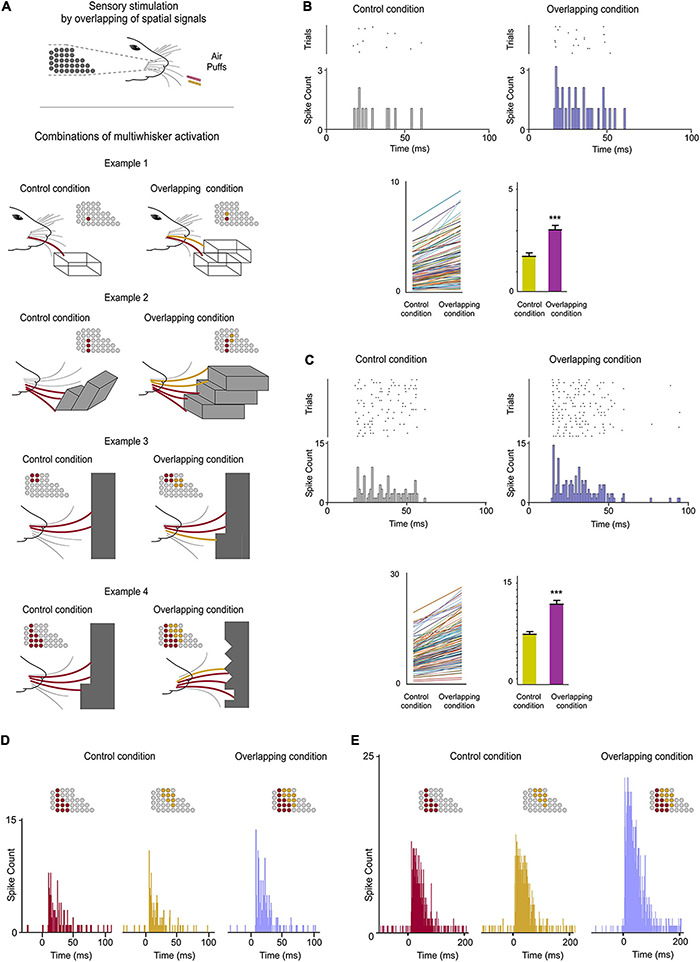
POm integration of spatial signals. **(A)** Sensory stimulation was produced by the application of individual (Control Condition) or simultaneous air puffs (Overlapping Condition) activating different whiskers and producing diverse combinations of multiwhisker activation. The number of whiskers stimulated by each air puff was varied to generate different combinations. They were repeated multiple times (range 20–70 repeats) and POm responses to them were studied and quantified by comparison between Control and Overlapping conditions. Four examples of these combinations are shown. The whiskers stimulated are depicted in different colors in the schematic representations of the whisker pads. Simulated objects and surfaces are only shown to illustrate similar real overlappings. **(B)** POm response magnitude increased when whiskers were activated simultaneously as can be appreciated in the peri-stimulus time raster plots and histograms of a representative POm single-unit response for 20 trials evoked by the example 2 in panel **(A)**. Data comparing the spike rate in Control and Overlapping conditions of single-units (*n* = 102, depicted in different colors) across stimulation combinations and the total mean response magnitude in both conditions are also shown. The mean firing rate of all single-units was significantly increased in the Overlapiing condition (72%; *p* < 0.001; Wilcoxon matched-pairs test). **(C)** Same as in panel **(B)**, but for multi-unit activity. Raster plots and PSTHs of a demonstrative POm multi-unit response for 40 trials evoked by the example 3 in panel **(A)**. The spike rate in Control and Overlapping conditions of multi-units (*n* = 136, depicted in different colors) across stimulation combinations and the total mean response magnitude in both conditions are shown. The mean firing rate of all multi-units was significantly increased in the Overlapiing condition (56%; *p* < 0.001; Wilcoxon matched-pairs test). **(D)** PSTHs of POm single-unit responses evoked by the example 4 in panel **(A)** (40 trials). To visualize the facilitative integration of overlapping spatial signals, individual evoked responses in the Control Condition are displayed separately. **(E)** Same as in panel **(D)**, but for multi-unit activity.

**FIGURE 4:**
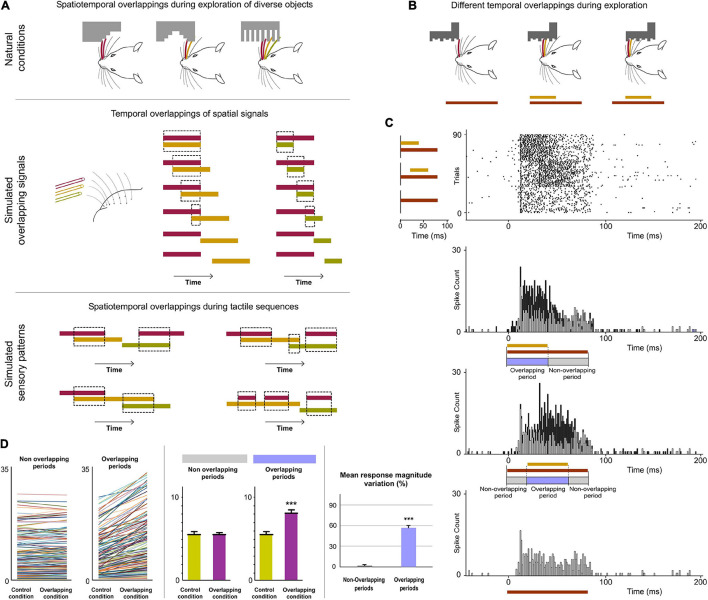
POm integration of spatiotemporal overlapping dynamics. **(A)** Sensory patterns can be simulated by the simultaneous activation of different whiskers and by varying the number of whiskers stimulated, their combination and the order and duration of air pulses to produce different spatiotemporal overlappings. A variety of spatiotemporal patterns were designed to simulate possible real stimuli or sequences of stimuli using controlled multiwhisker deflections performed by overlapping air puffs of different durations applied to different whiskers including neighboring whiskers or whiskers farther apart across the whisker pad. The duration of air puffs and the temporal overlapping between them were varied to generate different combinations (protocols). The number of whiskers stimulated by each air puff was varied to generate different sensory patterns using the same protocol. Some examples are shown. Air puffs are represented by color lines. Their length reflects the duration of the signal. Temporal overlappings between sensory signals are highlighted. **(B)** Schematic illustration of a simulated tactile sequence reflecting the generation of different temporal overlappings. **(C)** Raster plots and PSTHs of representative POm responses evoked by temporal overlappings illustrated in the tactile sequence in panel **(B)** are shown. The appearance of a new signal during the presence of an existing signal was integrated by POm. This was reflected in precise increases in POm activity. These increases in sustained responses were only produced during the overlapping time between them (Overlapping period) but not during the non-overlapping time (Non-overlapping period of response). The duration of the Overlapping and Non-overlapping periods is indicated. Note that the increases in POm activity during overlapping periods were sustained along the temporal overlapping. **(D)** Plots comparing the spike rate of all recorded multi-units (*n* = 155, depicted in different colors) during the Overlapping and Non-overlapping response periods in Control and Overlapping conditions across sensory patterns. The mean firing rate was increased in the Overlapping periods (*p* < 0.001; Wilcoxon matched-pairs test) but not in the Non-overlapping periods where the mean magnitude of responses did not change (*p* = 0.43; Wilcoxon matched-pairs test). Response magnitude variation (%) between Control and Overlapping conditions in Non-Overlapping and Overlapping periods (*p* < 0.001, Wilcoxon matched-pairs test) is also shown. ***P < 0.001.

**FIGURE 5:**
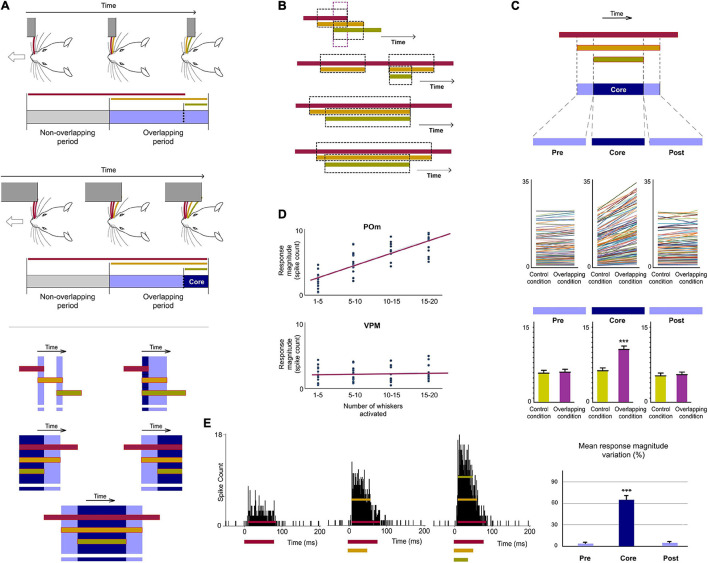
POm is highly sensitive to the dynamical spatiotemporal structure of sensory patterns and to the variability of their parts. **(A)** Multiple and constantly changing overlappings occur dynamically during contacts with objects and surfaces during active exploration. Accordingly, the number of whiskers implicated is constantly changing. As can be appreciated in these schematic illustrations of two simulated tactile sequences, their Overlapping periods are different. Defined by the number of whiskers simultaneously activated, “simple” overlappings (in light blue) and more “complex” overlappings (in dark blue) were used to understand how POm codifies this variability (bottom panel). The parts of the Overlapping periods with the mayor number of whiskers were selected and defined as “Core” parts. **(B)** Spatiotemporal patterns produced by the simultaneous application of a third air puff activating additional whiskers. Different spatiotemporal overlappings were generated by varying the onset and duration of the third air puff and the number of extra whiskers stimulated by this air puff. **(C)** POm responses during Overlapping periods were divided in three subperiods (Pre, Core, and Post). They were compared before (Control condition) and after the application of the third air puff (Overlapping condition). Data comparing the spike rate of all recorded multi-units (*n* = 101, depicted in different colors) in these conditions during Pre, Core, and Post subperiods across sensory patterns and the total mean response magnitude in both conditions in these subperiods are described. The mean firing rate was increased in the Core subperiod (*p* < 0.001; Wilcoxon matched-pairs test) but not in the Pre (*p* = 0.63; Wilcoxon matched-pairs test) and Post (*p* = 0.74; Wilcoxon matched-pairs test) subperiods. **(D)** Correlation between POm response magnitude and the number of whiskers simultaneously overlapped. Data from VPM are also shown for comparison. POm: Pearson correlation coefficient, *r* = 0.79, *p* < 0.001, *n* = 51 multi-units in 10 rats; VPM: *r* = 0.19, *p* < 0.001, *n* = 45 multi-units in 8 rats. The number of whiskers simultaneously activated is computed by POm. Note the profound functional difference between these nuclei. **(E)** PSTHs for a representative example showing that POm response magnitude in Overlapping periods gradually increased as more whiskers were temporally overlapped. Note that the temporal structure of these different sensory events is reflected in their corresponding POm responses. ***P < 0.001.

Sensory stimulation was characterized by spatiotemporal patterns of multiwhisker deflections simulating possible real stimuli or sequences of stimuli. Details of the multiwhisker stimulation patterns are described in Figures 2A, 3A, 4A, 5A,B. Anesthetized rats were used to facilitate their application. Using a pneumatic pressure pump (Picospritzer) that delivers air pulses through polyethylene tubes (1 mm inner diameter; 1–2 kg/cm^2^), sensory patterns were generated using controlled multiwhisker deflections performed by overlapping air puffs of different durations (20–2000 ms) applied to different whiskers in one or both sides of the face and avoiding skin stimulation. Accordingly, many whiskers were activated simultaneously producing different spatiotemporal overlapping dynamics. The air-puffers were precisely placed and the whiskers were trimmed to a length of 10–30 mm to allow precise overlapping stimulations. To simulate natural stimuli, overlappings produced by the activation of whiskers in different directions were also included. A variant order was adopted for delivering the stimulation patterns to avoid possible temporal dependency. We applied 20–70 trials per pattern at low frequency (0.3–0.5 Hz). Receptive field sizes were determined by deflecting individual vibrissae with a hand-held probe and monitoring the audio conversion of the amplified activity signal.

### Inactivation and Lesion of Thalamic Nuclei and Cortical Areas

We inactivated the cortex with local infusions of lidocaine (1%) or muscimol (1 mg/ml). To evaluate the proper level of cortical inactivation, the basal activity and sensory responses to whisker stimulation were continuously checked in the inactivated cortical area. Infusions were repeated every 15 min until cortical activity recorded in deep layers was silenced, on average 25 min after the first application. Pharmacological deactivation of POm was performed by injecting 100–200 nL of muscimol (1 mg/ml) in this thalamic nucleus. The drug was slowly delivered through a cannula connected to a Hamilton syringe (1 μl) over a one-minute period.

Lesions of different cortical areas were also performed in our experiments. To assure the precision of cortical lesions, the skull was exposed and openings were precisely restricted to the corresponding cortical area according to stereotaxic coordinates [S1, described above; secondary somatosensory cortex (S2), P 0–3.7; L 5.5–7.5; primary motor cortex (M1), A 0.5–2.5, L 0.2–3]. Lesions were made by cutting and aspirating the cortical tissue and included superficial and deep layers of these areas.

### Histology

After the last recording session, animals were deeply anesthetized with sodium-pentobarbital (50 mg/kg i.p.) and then perfused transcardially with saline followed by formaldehyde solution (4%). After perfusion, brains were removed and post-fixed. Serial 50 μm-thick coronal sections were cut on a freezing microtome (Leica, Germany). These sections were then prepared for Nissl staining histochemistry for verification of electrodes tracks, delimitation of cortical lesions and discrimination of thalamic nuclei. Positions of the electrode tips and extensions of cortical lesions were histologically verified by comparing these coronal brain sections with reference planes of the rat brain stereotaxic atlas (Paxinos and Watson, 2007).

### Data Acquisition and Analysis

Data were recorded from PrV, SpVi, VPM, POm, and S1. The raw signal of the *in vivo* extracellular recordings was filtered (0.3–300 Hz for local field potentials and 0.3–5 kHz for units), amplified (DAM80 preamplifier, WPI) and digitalized (Power 1401 data acquisition unit, CED, United Kingdom). We applied a semi-automatic spike sorting technique (template-matching) provided by the Spike2 software (CED). Threshold crossing events were used to compute templates of spike waveforms which were subsequently used to assign individual spikes. To control for single unit separation, we applied principal component analysis (PCA) of the detected waveforms. Single units had to show cluster separation after plotting their first three principal components. Furthermore, we plotted inter-spike interval (ISI) distributions of the units, which was allowed to be only above 1 ms. Multi-units were collected by amplitude sorting. Local field potentials were obtained and a short-time Fourier transform was computed using MATLAB to construct a color-coded spectrogram.

To quantify the spontaneous activity, the number of spikes occurring in the 100 ms preceding the stimulus was counted. We defined response magnitude as the total number of spikes per stimulus occurring between response onset and offset from the peristimulus time histogram (PSTH, bin width 1 ms unless noted otherwise). Response onset was defined as the first of three consecutive bins displaying significant activity (more than three standard deviations above the mean spontaneous activity) after stimulus and response offset as the last bin of the last three consecutive bins displaying significant activity. Response duration was defined as the time elapsed from the onset to offset responses. In all figures, raster plots represent each spike as a dot and each line corresponds to one trial. Spikes were aligned on stimulus presentation (Time 0 ms). The temporal interval between the trigger command and whisker deflection following air puff was calculated (quantified in ∼9 ms; tube placed at ∼10 mm from the whiskers) and used as the reference onset time for whisker deflection following the air puff trigger command. This delay was corrected during analysis.

All data are expressed as the mean ± standard error of the mean (SEM). Error bars in the figures correspond to SEM. For normally distributed data (Shapiro-Wilk normality test), statistical analyses were conducted using a Student’s *t*-test. Non-normally distributed data were evaluated using a Wilcoxon matched-pairs test. Multiple comparisons were evaluated using a One-way ANOVA test. *Z*-scores were computed relative to the activity across all bins in the PSTH from −100 to 400 ms respect to stimulus onset. These computations and plots were done in MATLAB. Statistical significance was considered at ^∗^*P* < 0.05, ^∗∗^*P* < 0.01, ^∗∗∗^*P* < 0.001.

## Results

### Sustained Activity in POm

Whisker-evoked responses in POm were examined by *in vivo* extracellular recordings from rats in a lightly anesthetized state using stimuli with different durations within the range used by these animals during their natural explorations (Figures 1, 2). While studying multi-unit activity, we found that this nucleus was able to generate sustained responses even to long-duration stimuli. This capacity was consistent across all animals (*n* = 12). The duration of the stimulus did not alter the mean onset latency of responses (11.12 ± 0.60 ms, ranged from 7 to 16 ms, *n* = 119 multi-units, *p* = 0.52, One-way ANOVA; Figure 2D). To study how this multi-unit activity replicated the response profile of individual POm neurons, we also characterized this phenomenon studying POm single-units (*n* = 91). We found that POm neurons responded tending to generate sustained responses. This indicated that the population response formed by the combination of spikes from these neurons allowed for the encoding of stimuli duration by POm sustained activity (Figure 2B). Thus, we mainly used POm multi-unit responses for further analyses.

Since POm receives strong sensory projections from the trigeminal complex (Chiaia et al., 1991; Veinante et al., 2000a; El-Boustani et al., 2020), whisker-evoked responses were also investigated in the principal (Pr5; *n* = 47 multi-units) and in the interpolar (SpVi, *n* = 52 multi-units) trigeminal nuclei (11 rats). Sustained responses were only found in SpVi. The mean onset latency of these sustained responses was 8.31 ± 0.57 ms (ranged from 5 to 11 ms).

These results show that POm has the capacity to sustain its activity to encode and represent tactile input duration with high accuracy (Castejon et al., 2016). Given that tactile inputs are usually longer than the duration of multiple whisk cycles, this capacity could be used to codify sensory patterns and sequences of stimuli. The following experiments were designed to study the implication of POm in the encoding of these phenomena.

### POm Spatial Integration of Multiwhisker Stimulation

During whisking rats integrate signals from many whiskers to obtain accurate tactile information from their environment. Our next experiments were designed to map how sensory information, produced when multiple whiskers are activated simultaneously during a tactile event, is encoded in the activity of POm. First, we characterized the response properties of POm to simple spatial overlapping stimuli precisely delivering simultaneous air-puffs to different whiskers at different locations across the whisker pad. Consistent with published data (Diamond et al., 1992; Ahissar et al., 2000), we found large multiwhisker receptive fields (mean receptive field size: 10.9 ± 3.1 whiskers; *n* = 42 units). We observed that POm responses exhibited increases in firing rate when different whiskers were activated simultaneously. To investigate this POm capacity in detail, we studied the integration of signals by POm across a variety of reproducible spatial overlapping combinations (Figure 3A). Across them, we found that POm multi-unit responses showed increases in response magnitude during overlappings of spatial signals (quantified in Figure 3C). This was produced by a facilitative integration of overlapping signals (Figures 3D,E). The spatial integration was also observed between remote whiskers. The mean onset latency and duration of POm responses to different signals were not changed by their overlapping (Figure 3C). We also investigated these effects studying POm single-units (quantified in Figure 3B). These findings were consistent across all animals (*n* = 15). Importantly, the robustness of this capacity of encoding seemed to be obtained from population response (Figure 3C). Accordingly, we used POm multi-unit responses for further analyses of POm integration.

### POm Encoding of Spatiotemporal Overlapping Dynamics

Next, since tactile events typically have spatiotemporal structures that change dynamically in time, we studied how spatial integration (spatial dimension) occurs throughout the duration of the sensory pattern (temporal dimension) in 18 rats (Figure 4). Interestingly, when overlappings of spatial signals were produced by delivering simultaneous air-puffs with different durations or with the same duration but applied at different times (Figure 4A middle panel), increases in sustained responses were only observed during the overlapping time between them (data showing the quantification of this effect are described in Figure 4D). The spatial integration of multiwhisker activation was only produced during the temporal overlapping. Importantly, the increases of POm activity were sustained along the temporal overlappings (Figure 4C). Therefore, the time shared by overlapping signals is also encoded by POm activity.

Then, we studied these effects using sensory patterns formed by diverse temporal overlappings (Figure 4A bottom panel). We designed these patterns to simulate possible real stimuli or sequences of stimuli similar to those occurring in natural circumstances, such as sequential activation of multiple whiskers with partial temporal overlapping between them, simultaneous and delayed activation of different whiskers with different durations producing sequences with diverse spatiotemporal overlappings and precise sequences of long sustained activation of several neighboring whiskers overlapped with repetitive brief stimulations of remote whiskers. Across patterns, precise changes in POm sustained activity consistent with the spatiotemporal structure of the patterns were observed. Accurate increases in POm sustained activity were produced during the overlapping time between spatial signals reflecting changes in the spatiotemporal structure of sensory patterns.

Additionally, since in natural conditions the spatiotemporal structure of sensory patterns changes dynamically, the number of whiskers simultaneously implicated is not homogeneous along their duration. As can be appreciated in Figure 5A, dissimilar overlappings of spatial signals can produce sensory patterns formed by different parts with diverse complexities. To investigate the capacity of POm to represent these parts and to obtain a better quantification of this form of encoding, more sensory patterns were generated by overlapping additional spatial signals and by varying their onset and duration (Figure 5B). We selected the part with the mayor number of whiskers (“Core”) in the overlapping period of each pattern and divided the POm response into three subperiods: Pre, Core and Post. Precise POm activity changes were found during these different times of POm response reflecting the diversity between parts along the patterns. Across them, POm activity was significantly increased in the Core subperiod compared to Pre and Post subperiods (described and quantified in Figure 5C). This demonstrated that when additional signals were temporally overlapped, POm codified this by increasing its activity only during the temporal presence of these signals. We found that POm response magnitude in all overlapping periods gradually increased as more inputs were temporally overlapped in the patterns (Figures 5D,E). Together, these results showed that the dynamical spatiotemporal structure of sensory patterns and the different variety of their parts was accurately reflected in precise POm activity fluctuations. Importantly, we observed that POm generated very similar patterns of integrated activity when different whiskers were activated by the same stimulation protocol. This finding is in agreement with the less accurate somatotopy of the nucleus and suggests that the function of POm integration is not the combined representation of specific whiskers but the encoding and extraction of generic sensory patterns from the entire vibrissal array.

### No Sustained Activity in VPM. Stimuli Overlapping Did Not Alter Whisker Responses in VPM

**FIGURE 6:**
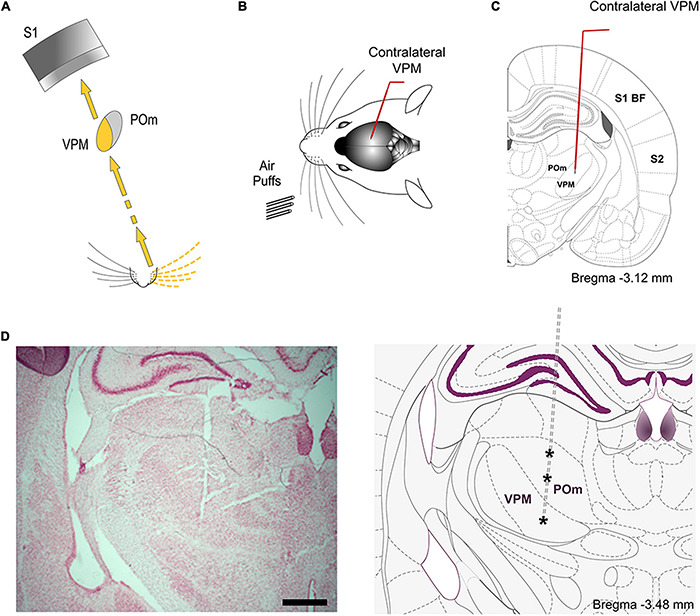
VPM. **(A)** Schematic illustration of the lemniscal pathway. **(B)** Schematic drawing displaying the sensory stimulation *via* patterns of multiwhisker deflections. Recordings were made in the contralateral VPM nucleus. **(C)** Coronal section illustrating a recording electrode inserted into the VPM. **(D)** The left panel shows a representative Nissl stained coronal section displaying the location of the sequence of recording sites (indicated by asterisks in the other panel) in POm and VPM and the track left by the electrode. An atlas schematic reconstruction of this coronal section is shown in the right panel (Paxinos and Watson, 2007). Scale bar, 1 mm.

**FIGURE 7:**
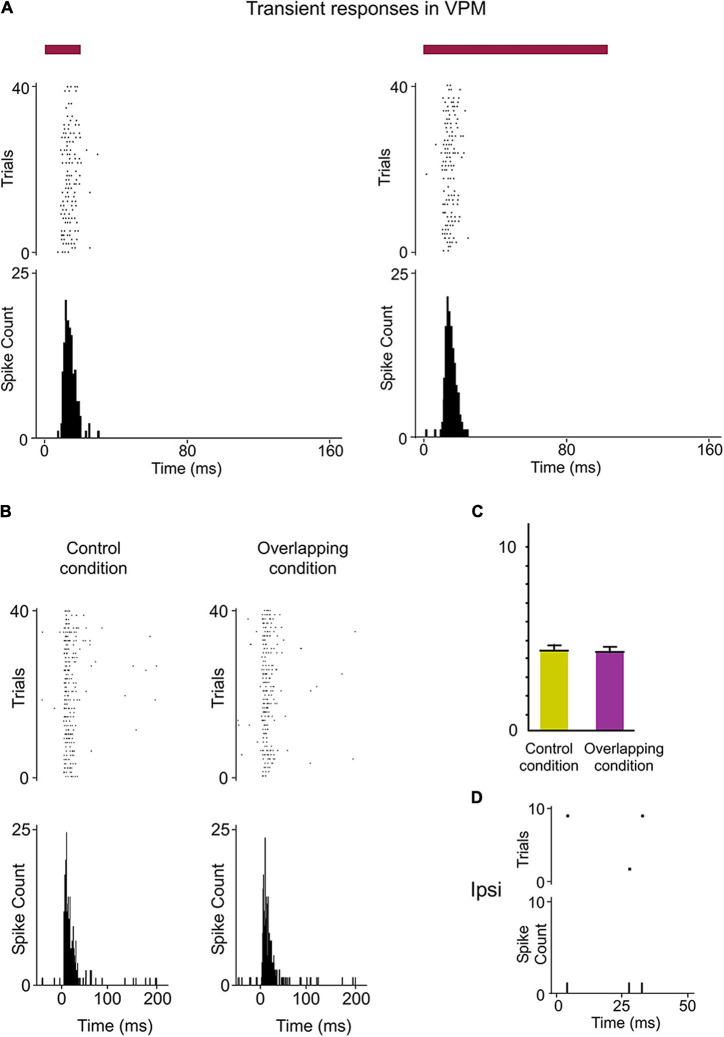
Response modes differed drastically between VPM and POm. **(A)** VPM responses were not sustained along stimulus presence. They were transient responses just to the onset of stimuli. Raster plots and PSTHs showing multi-unit VPM transient responses evoked by the same stimulus with different durations (20 and 100 ms). Note that VPM responses do not allow the discrimination between them. Red color lines indicate the duration of the stimulus. **(B)** Raster plots and PSTHs showing that VPM response to simultaneous multiwhisker activation (Overlapping condition) is very similar to individual whisker activation alone (Control condition). **(C)** The mean response magnitude across multi-units (*n* = 96) recorded in VPM did not change by the overlapping of spatial signals (−2%, *p* = 0.17, Wilcoxon matched-pairs test). The mean onset latency of VPM responses was 8.32 ± 0.31 ms (ranged from 6 to 11 ms). **(D)** VPM did not respond to ipsilateral stimuli.

To complement the analyses described above, whisker-evoked responses in VPM were examined using stimuli with different durations (Figure 6). Consistent with published data (Diamond et al., 1992), VPM responses showed high spatial resolution (mean receptive field size: 2.3 ± 0.7 whiskers; *n* = 44). In contrast to POm responses and in agreement with previous findings (Castejon et al., 2016), VPM responses did not show sustained response patterns. Therefore, response modes differed drastically between these nuclei. POm was persistently activated during whisker stimulation, whereas VPM was only transiently activated at the onset of stimuli (Figure 7A). Long stimuli evoked an onset response at the beginning of the stimulus and, occasionally, an offset response at the end but we did not find sustained responses during stimulus presence in VPM.

Since VPM responses were transient lasting tens of milliseconds, they seem to be excessively short for integrating over multiple whisks or longer sensory events. This suggests that the temporal integration in VPM is comparatively weak. To corroborate this, we characterized VPM responses delivering the same spatiotemporal patterns of multiwhisker activation used to characterized POm responses. Consistently across animals (*n* = 15), we did not find a significant change of VPM responses by multiwhisker stimuli application (Figures 7B,C, see also Figure 5D). This is in agreement with previous findings showing that VPM response to simultaneous multiwhisker activation is very similar to individual whisker activation alone (Aguilar and Castro-Alamancos, 2005).

Together, our results show that VPM responses are different from those of POm and suggest significant functional differences between POm and VPM thalamic nuclei in the processing of sensory patterns.

### POm Responses to Ipsilateral Whisker Stimulation

**FIGURE 8:**
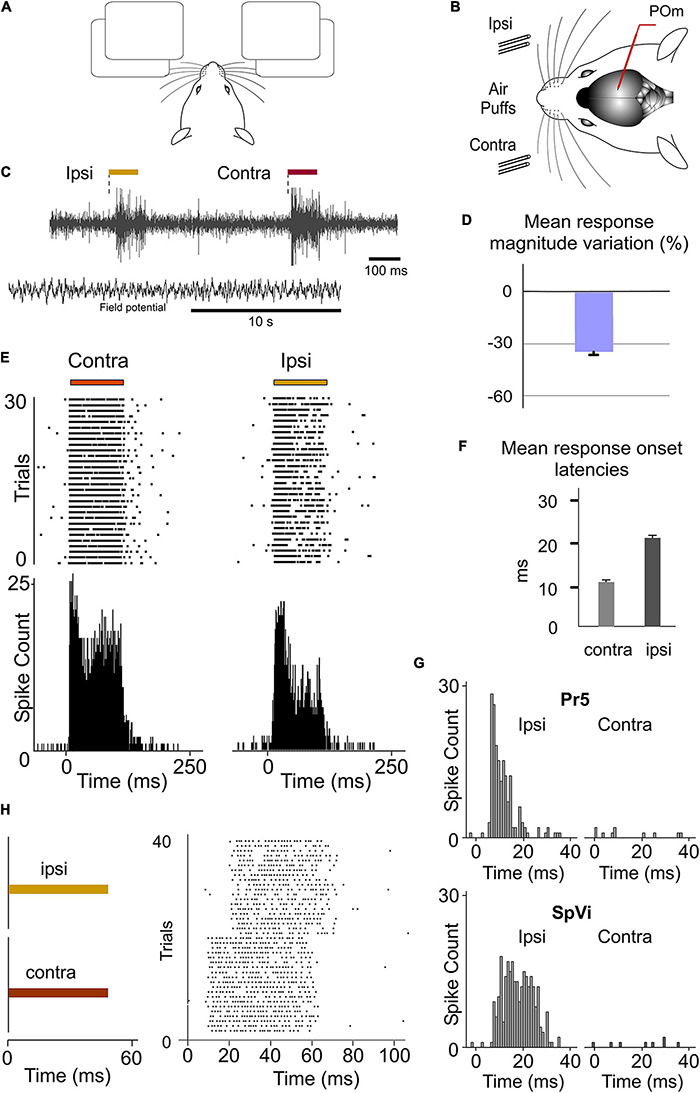
POm responses to tactile stimulation of ipsilateral whiskers. **(A)** Simulated illustration representing the use of bilateral information. **(B)** Schematic drawing displaying the sensory stimulation *via* patterns of contra-, ipsi-, or bilateral multiwhisker deflections. Recordings were made in POm. **(C)** Evoked activity produced by ipsi- and contralateral stimuli from the same recording in POm during the lightly anesthetized state. Field potential activity recorded in this state is also shown. **(D)** Mean ipsilateral response magnitude was significantly less than contralateral one (*p* < 0.001, Wilcoxon matched-pairs test, *n* = 90 multi-units). **(E)** Raster plots and PSTHs showing sustained POm responses evoked by contra- and ipsilateral multiwhisker stimulation. **(F)** Mean onset latencies of contra- and ipsilateral responses (*n* = 90 multi-units). The onset latency of ipsilateral responses ranged from 18 to 35 ms. **(G)** Evoked responses to contralateral whisker stimulation were not found in the Pr5 and SpVi trigeminal nuclei. Sustained responses to ipsilateral stimulation were only found in SpVi. **(H)** POm responses also lasted the duration of the ipsilateral stimulus.

Since during tactile exploration whiskers are usually stimulated bilaterally, it is then possible that POm could be implicated in the integration of bilateral signals. To test this hypothesis, we recorded POm responses to contralateral and ipsilateral stimuli during the lightly anesthetized state. Strikingly, we found that POm was also able to respond to tactile stimulation of ipsilateral whiskers (Figure 8). Our experiments showed multiwhisker ipsilateral receptive fields (mean receptive field size: 9.3 ± 2.7 whiskers; *n* = 42) and demonstrated that POm is not only characterized by broad contralateral receptive fields but also by broad ipsilateral ones. Across recorded multi-units (*n* = 90), the ipsilateral responses were weaker in magnitude than contralateral responses (Figures 8C–E) and longer in latency (mean response onset latency: 22.31 ± 1.28 ms versus 11.36 ± 0.82 ms). The difference between ipsi- and contralateral response onset latencies (∼10 ms, Figure 8F) suggests that ipsilateral sensory information is mediated by a different pathway. In agreement with this, we did not find evoked responses to contralateral whisker stimulation in the Pr5 and SpVi trigeminal nuclei (11 rats; Figure 8G), therefore POm responses to ipsilateral whisker stimulation were not driven by ascending peripheral activity conveyed directly *via* the trigeminal complex.

Next, using air-puffs that varied in duration, we found that POm has also the capacity to sustain its activity to encode and represent tactile input duration of ipsilateral stimuli. Although the ipsilateral response was weaker in magnitude, the capacity to codify the duration of the stimulus remained robust (Figures 8E,H). The duration of the ipsilateral stimuli did not alter the mean onset latency of responses (One-way ANOVA, *p* = 0.79). These findings were consistent across all animals (*n* = 10).

### POm Integration of Overlapping Ipsilateral Signals

**FIGURE 9:**
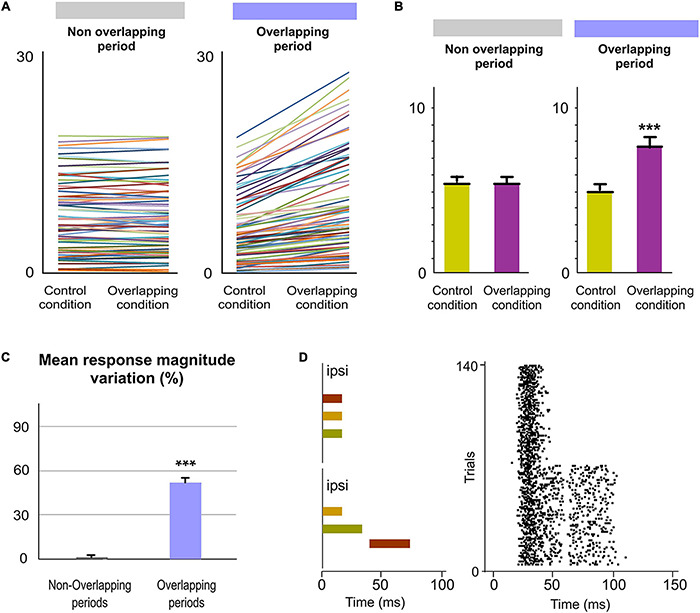
POm integration of ipsilateral overlapping events. **(A)** Data showing the quantification of the facilitative integration during overlappings of ipsilaterally evoked signals. Plots comparing the spike rate of all recorded multi-units (*n* = 80, depicted in different colors) during the Overlapping and Non-overlapping response periods in Control and Overlapping conditions across sensory patterns. **(B)** The mean firing rate was significantly increased in the Overlapping periods (*p* < 0.001; Wilcoxon matched-pairs test) but not in the Non-overlapping periods where the mean magnitude of responses did not change (*p* = 0.97; Wilcoxon matched-pairs test). **(C)** Mean response magnitude variation (%) between Control and Overlapping conditions in Non-Overlapping and Overlapping periods (*p* < 0.001, Wilcoxon matched-pairs test). **(D)** Raster plots of representative POm responses to two different overlapping protocols of multiwhisker ipsilateral stimulation. An increase in POm response magnitude can be appreciated as more inputs were temporally overlapped in these protocols. Note that the general temporal structure of the sensory pattern, the temporal position of its components (depicted in different colors) and their corresponding overlappings are reflected in the structure of POm response. ***P < 0.001.

Next, we investigated the implication of POm in the encoding of ipsilateral stimuli or patterns of stimuli. Accordingly, we studied POm responses to ipsilateral multiwhisker stimulation protocols producing different sensory patterns of spatiotemporal overlappings. Across these patterns, the firing rate was significantly increased in the Overlapping periods (Figure 9). These increases were also sustained along the temporal overlappings (Figure 9D). However, in the Non-overlapping periods, the magnitude of responses did not change. Again, we found precise changes in POm activity consistent with the spatiotemporal structure of the ipsilateral patterns (Figure 9D). These findings were consistent across all animals (*n* = 11).

We also characterized VPM thalamic responses delivering the same spatiotemporal patterns of ipsilateral multiwhisker activation in 7 rats. In contrast to POm, VPM did not respond to ipsilateral stimuli or to ipsilateral overlapping protocols (Figure 7D). Since the integration of tactile information from the two sides of the body is fundamental in bilateral perception, our results suggest a different implication of these thalamic nuclei in this function.

### Transmission of Integrated Sensory Activity Between Both POm Nuclei

**FIGURE 10:**
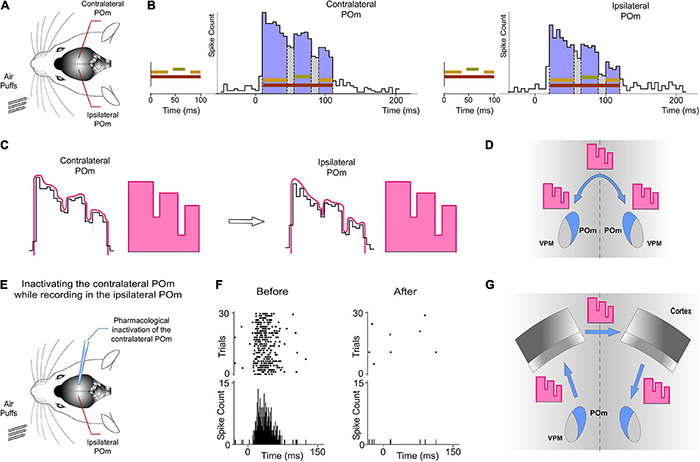
Ipsilateral sensory information is received by one POm from the other POm. This activity was transmitted preserving its integrated structure across the POm-POm loop. **(A)** Unilateral overlapping sensory patterns were applied while extracellular recordings were performed in both POm nuclei simultaneously. **(B)** POm responses evoked by these patterns showed different onset latencies but similar integrated structure in the contra- and ipsilateral POm nuclei. As can be appreciated in these representative PSTHs (bin width 5 ms) of POm responses, precise changes in POm activity caused by the integration of overlapping signals were similar in the contra- and ipsilateral POm nuclei. Note that the onset latency was longer in the ipsilateral POm. **(C)** PSTHs in B show that precise changes (fluctuations represented by lines in pink) in POm sustained activity caused by the integration of overlapping stimuli were precisely conserved. Therefore, these “structured patterns” of integrated information encoded by POm (represented by schematic patterns in pink) were interhemispherically transmitted to the other POm preserving their integrated structure **(D)**. **(E)** POm responses to ipsilateral stimulation were studied before and after pharmacological deactivation by muscimol (1 mg/ml) injection in the opposite POm. **(F)** POm responses to ipsilateral stimulation were abolished when the opposite POm was pharmacologically deactivated. **(G)** The latencies suggest that the POm-POm loop could be formed by a thalamocortical-callosal-corticothalamic route.

Our findings raised the question of by which route(s) is the ipsilateral information relayed to POm. The delay (10 ms) that we observed for ipsilateral information suggested an indirect transfer of ipsilateral information between hemispheres from the other POm. To test this possibility, unilateral overlapping sensory patterns were applied while extracellular recordings were performed in both POm nuclei simultaneously in 6 rats (Figure 10). We found that responses evoked by these patterns were similar in both nuclei. In agreement with data described above, they showed different onset latencies (Figure 10B) suggesting that evoked activity first arrives at the contralateral POm and is then transferred to the ipsilateral POm in the other hemisphere. This would indicate that both POm nuclei are indirectly connected forming a POm–POm loop.

Importantly, precise changes in POm activity caused by the integration of overlapping stimuli reflecting the spatiotemporal structure of the sensory pattern were precisely conserved (Figure 10B). Therefore, these patterns of integrated information encoded by one POm were transmitted through the loop to the other POm preserving their integrated structure (Figure 10C).

To confirm that ipsilateral activity reaches one POm from the other POm, we pharmacologically deactivated one of them by muscimol (1 mg/ml) injection (Figure 10E). We found that evoked responses in the second POm to ipsilateral stimulation were abolished in the majority of cases (4 out of 6 rats; Figure 10F). However, they were almost abolished but not completely eliminated in 2 cases (−91%, *p* < 0.001). The residual activity in these cases may be attributed to an incomplete deactivation of the opposite POm.

Together, these findings demonstrated a transmission of integrated sensory activity between both POm nuclei.

### POm Integration of Bilateral Events

**FIGURE 11:**
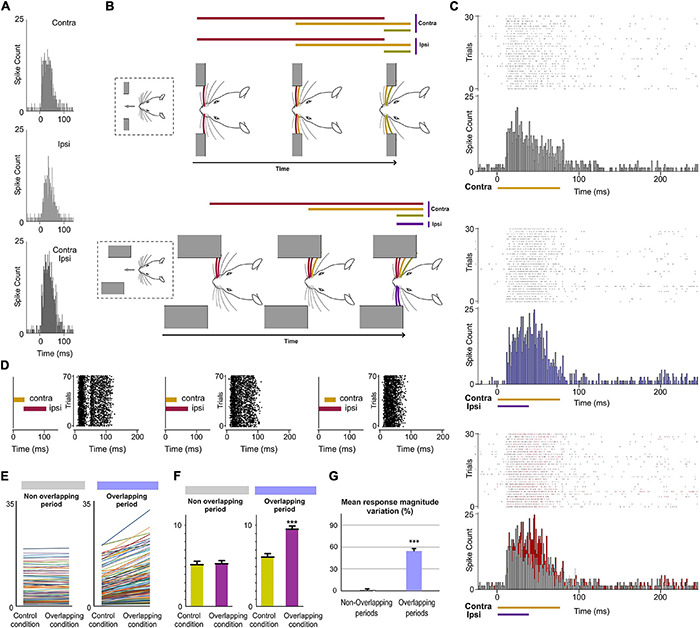
POm integration of bilateral events. **(A)** PSTHs of POm responses evoked by contra-, ipsi- and bilateral whisker stimulations (stimulus duration 40 ms; 30 trials shown for each stimulus). To visualize the facilitative integration of bilateral signals, contra- and ipsilateral evoked responses are displayed separately. **(B)** As can be appreciated in these two simulated tactile sequences, many whiskers on both sides are sequentially deflected simultaneously. In our experiments, bilateral sensory patterns were generated by the application of contralateral and ipsilateral stimuli activating different subsets of whiskers on both sides of the face at mirror (matched whiskers on each side) and non-mirror positions. **(C)** Raster plots and PSTHs showing demonstrative POm responses evoked by contralaterally (top) and bilaterally (middle) evoked overlapping stimulation using stimuli with different durations. Note that the time shared by these bilateral overlapping inputs is encoded by a precise increase in POm activity. This increment is depicted in red in the bottom panel. **(D)** The time interval between contralateral and ipsilateral stimuli was varied to produce different overlappings and to study their integration. Raster plots of POm responses to different bilateral overlapping stimulation protocols are shown. As can be appreciated in the left panel, no integration was observed when the contra- and ipsilateral stimuli were not temporally overlapped. **(E)** Data showing the quantification of the facilitative integration during overlappings of contralaterally and ipsilaterally evoked signals. Plots comparing the spike rate of all recorded multi-units (*n* = 187, depicted in different colors) during the Overlapping and Non-overlapping response periods in Control and Overlapping conditions across bilateral sensory patterns. **(F)** The mean firing rate was significantly increased in the Overlapping periods (*p* < 0.001; Wilcoxon matched-pairs test) but not in the Non-overlapping periods where the mean magnitude of responses did not change (*p* = 0.41; Wilcoxon matched-pairs test). **(G)** Mean response magnitude variation (%) between control and overlapping conditions in Non-Overlapping and Overlapping periods (*p* < 0.001, Wilcoxon matched-pairs test). ***P < 0.001.

Finally, since bilateral events occur concurrently producing different overlappings, the next question to investigate was whether POm would be able to codify these bilateral dynamics. It would require the precise integration of contralateral and ipsilateral sensory inputs. To investigate the implication of POm in these intricate computations, we applied spatiotemporal overlapping patterns of bilateral multiwhisker stimulation simulating possible real bilateral sensory events. We measured the responses of the nucleus to the overlapping patterns and found that POm precisely integrates tactile events from both sides (Figure 11). We found that precise changes in the spatiotemporal structure of bilateral events evoked different patterns of POm integrated activity. Across sensory patterns, the firing rate was increased in the Overlapping periods (quantified in Figure 11) but not during the non-overlapping time. This indicated that the time shared by overlapping ipsi- and contralateral stimuli was encoded by POm activity. These increases in firing rate were sustained along the Overlapping periods (Figure 11C). Similar effects were found stimulating identical (mirror) or different whiskers (non-mirror whiskers) on both sides. This is in agreement with the less accurate somatotopy of the nucleus and again indicates that the function of POm integration is not the combined representation of specific whiskers but the generic encoding of sensory patterns integrated from both whisker pads. Moreover, during Overlapping periods, facilitation of responses was found as more ipsilateral, contralateral or bilateral temporal overlapping inputs were added showing that the variability of the bilateral spatiotemporal overlappings was replicated in POm activity variations. These findings were consistently found across animals (*n* = 17).

Crucially, we found that the temporal interval between bilateral stimuli is critical to bilateral integration of sensory information. The delay (∼10 ms) that we observed for ipsilateral information determines the interaction between bilateral stimuli (Figure 11D). This is in agreement with previous findings showing that the activation of corticofugal projections from L5 in S1 by optogenetic stimulation increases ascending sensory responses within a well-defined time window (Groh et al., 2014).

### POm Nuclei Are Indirectly Connected Through the Cortex

**FIGURE 12:**
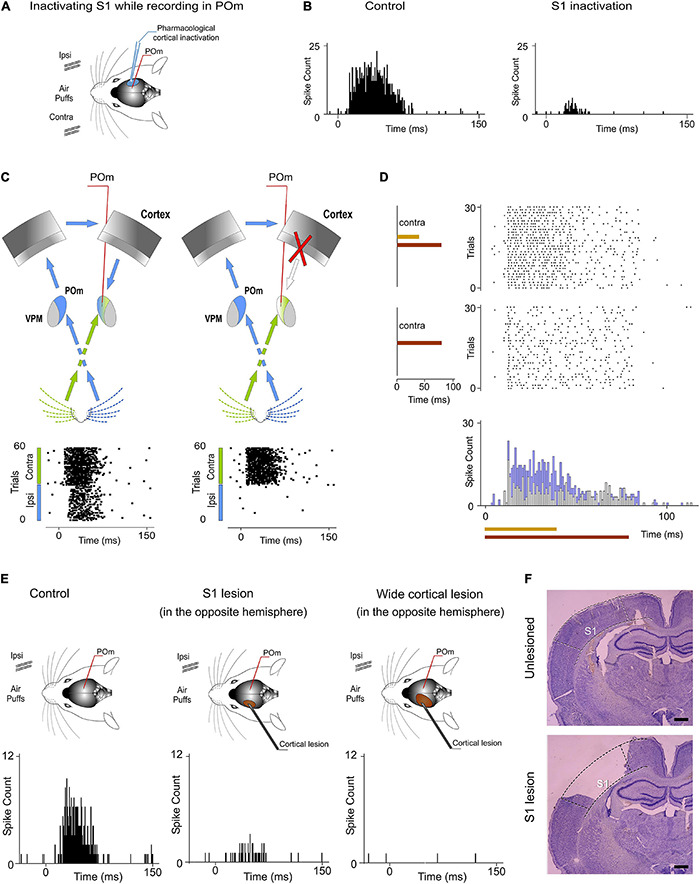
Ipsilateral and contralateral POm nuclei are indirectly connected through the cortex. **(A)** POm responses were recorded before and after S1 inactivation (*n* = 23 multi-units). **(B)** POm responses to ipsilateral whisker stimulation were almost abolished when S1 was inactivated or lesioned. PSTHs of representative POm responses before and after S1 inactivation. **(C)** Schematic illustration representing the transmission of contralateral and ipsilateral information to POm. As can be appreciated in the corresponding raster plots, POm response to ipsilateral whisker stimulation was eliminated when the POm-POm loop was interrupted by cortical removal. Note that POm response to contralateral stimulation was still present in this condition. **(D)** Cortical removal did not affect the capacity of POm to sustain its activity to codify stimuli duration and its capacity to integrate overlapping stimuli as can be appreciated in these POm responses recorded during this condition. **(E)** Representative POm responses in control, S1 lesion (in the opposite hemisphere) and wide cortical lesion (in the opposite hemisphere) conditions. An almost complete reduction but not a total elimination of ipsilateral responses was produced by the S1 lesion. POm responses to ipsilateral stimulation were completely eliminated only when a wider cortical extension was lesioned. **(F)** Example histology showing the removal of S1 from a lesioned animal. An unlesioned example is also shown for comparison. Scale bars, 1 mm.

Our results prompted us to examine the route(s) by which sensory information is transferred from one POm to the other. Cortical responses to ipsilateral whisker stimulation have been described in the somatosensory cortex (Shuler et al., 2001; Debowska et al., 2011). Therefore, ipsilateral activity seems to arrive at the contralateral POm by crossing the corpus callosum and descending from the cortex. Since POm receives strong innervation from corticofugal projection neurons in S1 (Veinante et al., 2000b), it is then possible that ipsilateral sensory stimulation could produce the activation of these descending corticofugal projections. This could have important implications on the integration of cortical inputs by POm and suggests that ipsilateral stimulation can be used to study the nature and content of the messages traveling through these corticofugal projections. To confirm that ipsilateral activity reaches POm *via* corticothalamic axons and to investigate whether these thalamic capacities are generated or mediated by cortical influence, we studied POm response properties before and after pharmacological deactivation of S1 (of the same hemisphere) by lidocaine (1%) or muscimol (1 mg/ml) application. As a control, we simultaneously recorded neuronal activity in the injected area. The electrodes were placed in the infragranular layer and the inactivation was confirmed by the absence of spontaneous and evoked activity. We found that POm responses to ipsilateral stimulation were almost abolished when S1 was inactivated (−89%, *p* < 0.001, paired *t*-test, *n* = 8 rats; Figure 12B). However, since the attempt to pharmacologically inactivate S1 can produce its partial deactivation and also can affect surrounding cortical areas, we confirmed our result by cortical lesion. The lesion was restricted to S1 and included superficial and deep layers of this area. This approach showed similar results. POm responses to ipsilateral stimulation were almost eliminated (−92%, *p* < 0.001, paired *t*-test, *n* = 6 rats).

POm also receives cortical projections from M1 and S2 (Alloway et al., 2008; Liao et al., 2010). However, we did not find a significant reduction of ipsilateral responses when only M1 and S2 were lesioned (−7%, *p* = 0.32, paired *t*-test, *n* = 4 rats). Together, these findings are in agreement with previous studies showing that cortical “driver” input (Sherman and Guillery, 1998) to POm originates almost exclusively from S1 (Veinante et al., 2000b).

When a wider cortical extension was lesioned including S1, M1, and S2, POm responses to ipsilateral stimulation was completely abolished in the majority of cases (4 out of 6 rats). However, we still found very small responses to ipsilateral stimulation in 2 cases (−94%, *p* < 0.001). Importantly, we found robust contralateral whisker-evoked responses in POm even when the cortex of the same hemisphere was inactivated. Cortical inactivation slightly affected the magnitude of POm responses to contralateral whiskers. We only found a minimal reduction of spikes (−8%; paired *t*-test, *p* = 0.06, *n* = 6 rats; Figure 12C) indicating that contralateral whisker-evoked responses were fundamentally driven by ascending activity. Furthermore, using air-puffs that varied in duration to stimulate contralateral whiskers, we found that cortical inactivation did not affect the capacity of POm to sustain its activity to codify stimuli duration (Figure 12D). We also measured POm responses to contralateral overlapping sensory patterns in this condition and found that cortical inactivation did not affect the capacity of POm to integrate overlapping stimuli (Figure 12D). On the basis of these results, we conclude that POm does not inherit these capacities from cortical influence.

Together, these results demonstrated that POm nuclei are indirectly connected through the cortex by showing that ipsilateral activity reaches POm *via* descending parallel corticofugal projections mainly from S1 of the same hemisphere. But, by which route(s) is the ipsilateral information relayed to S1? Since projections from POm to the cortex in the other hemisphere have not been described and since it is known that corticocortical transmission between hemispheres *via* callosal projections is the main route for ipsilateral sensory inputs (Shuler et al., 2001; Petreanu et al., 2007), it seemed that the POm-POm loop could be formed by a thalamocortical-callosal-corticothalamic route. To test this, we studied POm responses to ipsilateral stimulation before and after deactivation of S1 in the other hemisphere by lidocaine (1%) or muscimol (1 mg/ml) application. We found an almost complete reduction but not a total elimination of ipsilateral responses (−86%, *p* < 0.001, paired *t*-test, *n* = 6 rats). This was confirmed by S1 lesion (*p* < 0.001, paired *t*-test, *n* = 5 rats; Figure 12E). Again, when a wider cortical extension was lesioned including S1, M1, and S2, POm responses to ipsilateral stimulation were completely abolished in the majority of cases (4 out of 5 rats). We still found residual responses to ipsilateral stimulation in one case. It could be possible that the remaining activity in this case could be attributable to other cortical areas projecting to POm or to subcortical interhemispheric pathways such as the collicular commissure. These possibilities remain to be tested.

**FIGURE 13:**
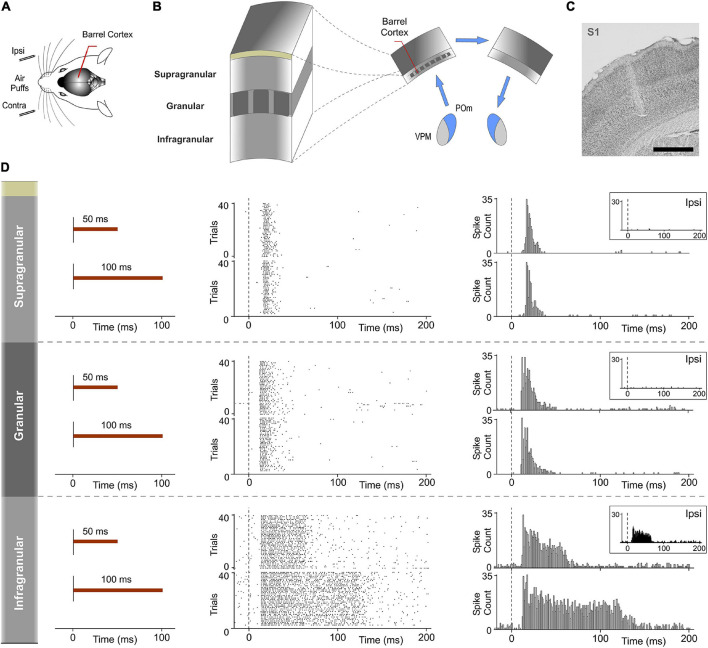
Response modes differed between cortical layers in S1. Sustained responses along the stimulus presence were found in the infragranular layer but not in granular and supragranular layers. **(A)** Recordings were made in the barrel cortex in S1 using ipsi- and contralateral stimuli with different durations. **(B)** Evoked responses in supra-, granular, and infragranular layers of the barrel cortex were examined to study the laminar implication in the processing of sustained activity and its transmission across the POm-POm loop. **(C)** Histological section displaying the location of the sequence of recording sites across cortical layers in S1 and the track left by the electrode. Scale bar, 1 mm. **(D)** Raster plots and PSTHs showing representative responses in supragranular, granular, and infragranular layers evoked by contralateral stimuli with different durations. The mean onset latencies of these responses were 15.42 ± 0.22 ms (ranged from 12 to 25 ms, *n* = 117), 11.81 ± 0.21 ms (ranged from 8 to 15 ms, *n* = 88), and 12.67 ± 0.34 ms (ranged from 8 to 16 ms, *n* = 102), respectively. Note that evoked responses in granular and supragranular layers were transient just to the onset of stimuli and that they do not allow the discrimination between different durations of the same stimulus. Responses to ipsilateral stimuli were only found in the infragranular layer (insets). The mean onset latency of these responses was 17.82 ± 0.55 ms (ranged from 16 to 28 ms, *n* = 36).

Finally, to investigate the implication of cortical layers in the processing and transmission of sustained activity between the thalamus and the cortex in the POm-POm loop, evoked responses across recorded multi-units in supra, granular and infragranular layers of S1 were examined using contralateral stimuli with different durations in 19 rats. Examination of the laminar profile of evoked activity across layers showed profound differences between them. We only found sustained responses lasting the duration of the stimulus in the infragranular layer (Figure 13). Similar to VPM responses, supra- and granular responses were only transiently activated at the onset of stimuli (Figure 13D). Long stimuli usually evoked an onset response at the beginning of the stimulus and an offset response at the end but we did not find sustained responses during stimulus presence in these layers. Moreover, we found that only the infragranular layer showed evoked responses to ipsilateral stimulation (Figure 13D). These results demonstrated different laminar implication in the processing of sustained activity and its transmission between hemispheres.

## Discussion

### Functional Significance of POm Capacities: POm Activity Fluctuations to Codify Patterns

Our findings show that POm capacities to sustain and integrate activity allow the representation of tactile events. Varying the spatiotemporal structure of sensory inputs, we found that POm is highly sensitive to sensory patterns in which the activation of individual whiskers strongly overlaps. Accurate increases in POm activity were produced during the overlapping time between spatial signals reflecting changes in the spatiotemporal structure of sensory patterns. Precise fluctuations of POm integrated activity seem to generate representations of these dynamics. This was produced by a facilitative integration of simultaneous signals. What could be the function of these precise thalamic activity fluctuations? Since rodents and other mammals have the ability to detect patterns embedded in a continuous stream of sensory activity, they may provide a mechanism for detecting spatiotemporal landmarks in the continuous flow of incoming sensory signals. They could be used to precisely decode sequence boundaries allowing for extraction of regularities and patterns from the flow of raw sensory information. Moreover, these fluctuations can also serve as relevant cues in sensorimotor adjustment, pattern recognition, perceptual discrimination and decision-making. From a functional perspective, active whisking and palpation movements can intentionally optimize the number, frequency and variation of overlappings to maximize the extraction of information (i.e., regularities) from objects, surfaces and textures during their exploration. Accordingly, adapting the active generation of precise overlappings of specific subsets of whiskers and their frequency would allow these animals to obtain the optimal resolution necessary to solve different perceptual or tasks requirements. In addition, VPM may provide the exact somatotopy to identify the specific whiskers activated in each fluctuation (see below). During active whisking, this may occur in every whisk cycle.

Across protocols, we found that varying the spatiotemporal structure of the sensory input produced different patterns of POm integrated activity. We observed that POm generates very similar patterns of activity (fluctuations) when different whiskers were activated by the same stimulation protocol. This finding is in agreement with the less accurate somatotopy of this nucleus and suggests that the function of POm integration is not the combined representation of specific whiskers but the encoding of generic sensory spatiotemporal patterns from the array of whiskers. Accordingly, our findings suggest that POm is a general encoder of tactile patterns. It is then possible that an important function of higher-order thalamic nuclei could be the encoding of patterns. This needs to be confirmed in other sensory modalities.

### POm Mediates Bilateral Sensory Processing

**FIGURE 14:**
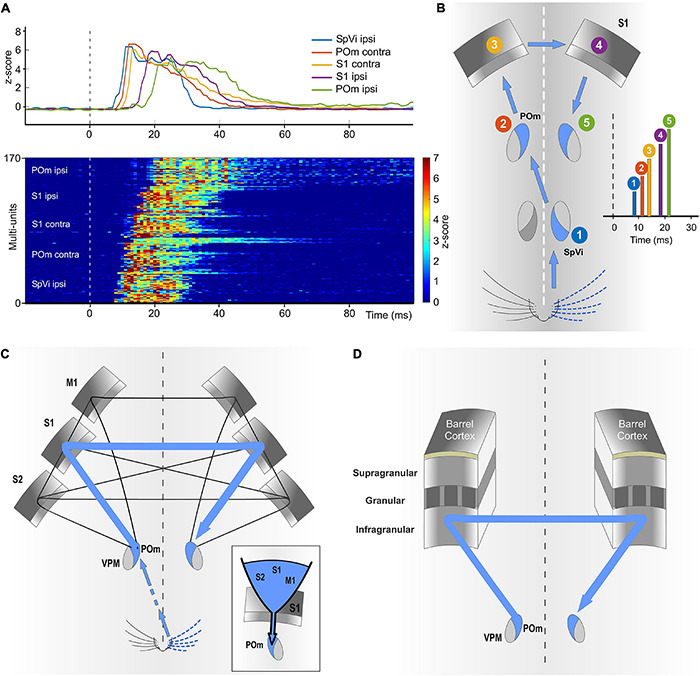
The POm-POm loop is formed by a functional network of parallel thalamocortical, interhemispheric, and corticothalamic projections. **(A)** Demonstrative responses to whisker stimulation in *z*-score across multi-units sequentially displayed along the POm-POm loop. The average responses are shown above. **(B)** Illustration recapitulating the mean onset latencies across the different brain structures implicated in the POm-POm loop. **(C)** Although different cortical areas are implicated, our results revealed that S1 plays a central role in this functional loop. The inset illustrates the idea that S1 may act as a funnel collecting activity from different areas and sending this information *via* corticothalamic projections to POm of the same hemisphere. This complex interhemispheric loop allows bilateral integration in the thalamus and is implicated in the bidirectional transmission of sustained activity between the higher-order thalamus and the cortex. **(D)** Our results indicate that the transmission of sustained activity across the POm-POm loop is supported by the infragranular layer. This loop could be also present in other sensory modalities.

Our results show that ipsilateral activity reaches one POm indirectly from the other POm (Figure 10). Moreover, these findings demonstrate a transmission of sensory activity between both nuclei through a functional POm-POm loop formed by thalamocortical, interhemispheric, and corticothalamic projections. We confirmed this interhemispheric pathway by inactivating different areas of the cortex in both hemispheres (Figure 12) and demonstrating that ipsilateral activity reaches POm mainly *via* S1 but not exclusively (Figures 14A–C). This suggests that POm nuclei are indirectly connected forming a complex network of parallel thalamocortical, interhemispheric, and corticothalamic projections. In agreement with this finding, it is anatomically well described that S1, MI, and S2 receive thalamocortical projections from POm (Ohno et al., 2012; El-Boustani et al., 2020), that they are, respectively, interhemispherically connected (Carvell and Simons, 1987; Kinnischtzke et al., 2014) and that they have corticothalamic projections to POm (Alloway et al., 2008; Liao et al., 2010). Moreover, POm is a strong driver of activity in S1, S2, and M1 (Theyel et al., 2010; Gambino et al., 2014; Casas-Torremocha et al., 2019; Zhang and Bruno, 2019; Gharaei et al., 2020) and bilateral sensory responses in S2 have also been described (Debowska et al., 2011).

The implication of different cortical areas was investigated revealing that S1 plays a central role in the POm-POm loop. Since S1 receives intra- and interhemispheric projections from these cortical areas (Carvell and Simons, 1987; Kinnischtzke et al., 2014), it is possible that S1 could collect activity from them (mostly from the other S1) and funnel this information *via* corticothalamic projections from layer 5 to POm (Figure 14A). This novel idea is in agreement with our results and with previous studies showing that cortical “driver” input (Sherman and Guillery, 1998) from layer 5 to POm originates almost exclusively from S1 (Veinante et al., 2000b). Moreover, it is known that motor and S2 cortical regions elicited strong direct input to L6b and L5 in S1 (Mao et al., 2011; Zolnik et al., 2020). This cortical architecture in which one specific area (S1 in the somatosensory system) acts as a functional funnel may be a common characteristic of the cerebral cortex and could be probably present in other sensory modalities.

Importantly, we found that cortical inactivation abolished POm responses to ipsilateral whiskers. However, cortical inactivation or lesion only minimally reduced POm responses to contralateral whiskers. This demonstrates that contralateral responses are mainly driven by input from subcortical sources whereas ipsilateral responses are driven by input from the cortex. This cortical input allows POm to obtain sensory information also from the ipsilateral part of the body. However, we found that VPM did not respond to ipsilateral stimuli. Unlike POm, VPM does not receive cortical input from L5 or L6b corticofugal projections of S1 (Hoerder-Suabedissen et al., 2018). Accordingly, the subcortical and cortical inputs allow POm to have sensory information from both sides of the body. This finding is central to functionally define and classify these thalamic nuclei.

### Interhemispheric Transmission of Integrated Activity Between Thalamic Nuclei

As far as we know, no studies had been performed to define the transfer of sustained activity through the thalamocortical-callosal-corticothalamic loops. These pathways have usually been studied separately. We investigated the nature and content of the activity carried by these projections and found that precise fluctuations in sustained activity caused by the integration of overlapping stimuli were precisely conserved (Figure 10). On the basis of this finding, we propose that these “structured patterns” of integrated information encoded by POm and transmitted through the loop preserving their integrated structure could have important functional implications in the representation of sensory information. They could be used as functional “templates” for different brain processes.

Since POm neurons have “multispecific” thalamocortical axons (Clascá et al., 2016) innervating several cortical areas including S1, S2, and M1 with different laminar profiles, our results suggest that these patterns of integrated activity generated by POm can be sent in parallel to different cortical targets. Therefore, the same message can be used by these areas and layers for different functions (i.e., perceptual, attentional, and motor). Moreover, since POm also projects to different brain structures including the amygdala, basal ganglia, insular, or ectorhinal cortex (Ohno et al., 2012; Smith et al., 2012), our results suggest that the same “templates” generated by POm can be used by these targets for diverse functions such as perceptual discrimination, familiarity, behavioral relevance, motivational meaning, or decision-making.

### Different Laminar Implication in the Processing and Transmission of Sustained Activity

The laminar analysis revealed that sensory-evoked responses in S1 had different temporal structures across layers (Figure 13). We only found sustained responses lasting the duration of the stimulus in the infragranular layer. However, not all responses in the infragranular layer were sustained. This in agreement with the complexity of this layer formed by different sublayers. Sustained responses were mostly observed in the superficial part of the infragranular layer corresponding to layer 5. In addition, similar to VPM responses, supra- and granular responses were only transiently activated at the onset of the contralateral stimuli but we did not find sustained responses along the stimulus presence. This is in agreement with previous findings that show higher sensory-evoked firing rates in L5 neurons (de Kock et al., 2007; Castejon et al., 2016) and sparse firing to sensory stimulation in supragranular layers of vibrissal cortex in S1 (de Kock et al., 2007; Petersen and Crochet, 2013; Clancy et al., 2015; Peron et al., 2015). Moreover, consistent with other studies (Shuler et al., 2001; Manns et al., 2004), responses to ipsilateral stimuli were only found in the infragranular layer (Figure 13). Together, these results indicate that the transmission of sustained activity across the POm-POm loop is supported by the infragranular layer (Figure 14D). This loop could be also present in other sensory modalities and animals (for example, a possible pulvinar-pulvinar loop in primates).

### POm Responses to Ipsilateral Stimulation, POm Sustained Activity and Its Interhemispheric Transmission Are Highly Sensitive to Anesthesia

During wakefulness or under light anesthesia, POm activity is significantly higher than during the deeply anesthetized state (Masri et al., 2008; Sobolewski et al., 2015; Zhang and Bruno, 2019). This suggests that normal POm functioning can be affected in this condition. Our findings are in agreement with this idea. In our experiments, we did not find POm responses to ipsilateral stimulation during the deeply anesthetized state (first hours after the application of urethane, 1.3–1.5 g/kg i.p.). These responses were only found during the lightly anesthetized state (Figure 1E). Moreover, supplementary doses of urethane abolished POm responses to ipsilateral whisker stimulation. These observations indicate that the transmission of sensory activity between hemispheres across the POm-POm loop could be highly sensitive to anesthesia. In agreement with this, it has been previously shown that increasing the level of anesthesia produces the elimination of evoked responses in S1 to ipsilateral stimulation (Armstrong-James and George, 1988; Shuler et al., 2001). This fact could explain why POm responses to ipsilateral stimulation had not been reported before. Together, this evidence indicates that high levels of anesthesia impair the real dynamics of POm functioning.

### Different but Complementary Functional Roles of VPM and POm. The Hypothesis of Complementary Components

As described above, two main parallel ascending pathways convey input from the whiskers to barrel cortex (Diamond et al., 1992; Veinante et al., 2000a). This anatomical segregation suggests a different functional role of these pathways and their corresponding thalamic nuclei in somatosensory processing. Our results showed that sensory stimulation protocols with similar spatiotemporal structures produced similar patterns of POm activity fluctuations even when different whiskers were activated by the same protocol. This suggests that accurate somatotopy is not a functional characteristic of this nucleus. Accordingly, we propose that to optimize the extraction of information from the sensory flow, the paralemniscal system must be complemented with an additional system providing precise somatotopy. This can be the functional role of the lemniscal pathway, phylogenetically more recent and characterized by a precise somatotopy (Diamond et al., 1992). Previous studies have shown that in this pathway the response properties of VPM neurons are very similar to those of PrV neurons (Chiaia et al., 1991). Our results, described here, are in agreement with this proposal showing important but functionally complementary differences between POm and VPM. This functional proposal which we have called the hypothesis of “Complementary Components” can explain why tactile information from whiskers is processed by parallel ascending pathways toward the cortex. This parallel architecture is also present in the majority of sensory systems in the brain and is conserved across animals. Accordingly, we propose that sensory systems have evolved to optimize the extraction of information from the environment and that the appearance of “complementary” pathways (as the somatosensory lemniscal pathway) during evolution was essential in that functional optimization.

In addition, our results demonstrate distinct laminar processing of the same stimulus by the cortex. They show that the content, type and nature of the messages that these layers receive, process and transfer are different. Therefore, different “Components” are also associated with distinct laminar profiles. They may play different but complementary functional roles. This could account for the different profiles of activity found in cortical layers (Figure 13D).

We propose that “Complementary Components” could be a basic principle of brain functioning. The nature (structured versus discrete), type (sustained versus transient), and content (integrated versus segregated) of neural activity processed and transmitted by different brain structures may determine their functional implication and must be differentiated.

## Data Availability Statement

The raw data supporting the conclusions of this article will be made available by the authors, without undue reservation.

## Ethics Statement

The animal study was reviewed and approved by Ethics Committee of the Autónoma de Madrid University and the Competent Spanish Government Agency (PROEX175/16), in accordance with the European Community Council Directive 2010/63/UE.

## Author Contributions

CC conceived the hypotheses, designed and conducted the experiments, analyzed the results, and wrote and edited the manuscript. JM-C conducted the experiments, analyzed the results, and reviewed the manuscript. AN designed and conducted the experiments, analyzed the results, and reviewed and edited the manuscript. All authors contributed to the article and approved the submitted version.

## Conflict of Interest

The authors declare that the research was conducted in the absence of any commercial or financial relationships that could be construed as a potential conflict of interest.

## Publisher’s Note

All claims expressed in this article are solely those of the authors and do not necessarily represent those of their affiliated organizations, or those of the publisher, the editors and the reviewers. Any product that may be evaluated in this article, or claim that may be made by its manufacturer, is not guaranteed or endorsed by the publisher.
